# Carrier–Tumor Cell Membrane Interactions for Optimized Delivery of a Promising Drug, 4(*RS*)-4-F_4t_-Neuroprostane

**DOI:** 10.3390/pharmaceutics15122739

**Published:** 2023-12-07

**Authors:** Ariana Abawi, Céline Thomann, Giovanna Lollo, Thierry Granjon, Emma Petiot, Anna Bérot, Camille Oger, Valérie Bultel-Poncé, Alexandre Guy, Jean-Marie Galano, Thierry Durand, Agnès Girard-Egrot, Ofelia Maniti

**Affiliations:** 1 Institute of Molecular and Supramolecular Chemistry and Biochemistry, ICBMS UMR 5246, University Lyon, Université Lyon 1, CNRS, F-69622 Lyon, France; ariana.abawi@etu.univ-lyon1.fr (A.A.); celine.thomann@univ-lyon1.fr (C.T.); thierry.granjon@univ-lyon1.fr (T.G.); emma.petiot@univ-lyon1.fr (E.P.); anna.brt@hotmail.fr (A.B.); agnes.girard-egrot@univ-lyon1.fr (A.G.-E.); 2 Laboratoire d’Automatique, de Génie des Procédés et de Génie Pharmaceutique, LAGEPP UMR 5007, University Lyon, Université Lyon 1, CNRS, F-69622 Lyon, France; giovanna.lollo@univ-lyon1.fr; 3 Pôle Chimie Balard Recherche, Institut des Biomolécules Max Mousseron, IBMM, UMR 5247, Université de Montpellier, CNRS, ENSCM, F-34293 Montpellier, France; camille.oger@umontpellier.fr (C.O.); alexandre.guy@umontpellier.fr (A.G.); jean-marie.galano@umontpellier.fr (J.-M.G.); thierry.durand@umontpellier.fr (T.D.)

**Keywords:** liposomes, drug delivery, membrane fluidity, 4(*RS*)-4-F_4t_-Neuroprostane

## Abstract

Nanomedicines engineered to deliver molecules with therapeutic potentials, overcoming drawbacks such as poor solubility, toxicity or a short half-life, are targeted towards their cellular destination either passively or through various elements of cell membranes. The differences in the physicochemical properties of the cell membrane between tumor and nontumor cells have been reported, but they are not systematically used for drug delivery purposes. Thus, in this study, a new approach based on a match between the liposome compositions, i.e., membrane fluidity, to selectively interact with the targeted cell membrane was used. Lipid-based carriers of two different fluidities were designed and used to deliver 4(*RS*)-4-F_4t_-Neuroprostane (F_4t_-NeuroP), a potential antitumor molecule derived from docosahexaenoic acid (DHA). Based on its hydrophobic character, F_4t_-NeuroP was added to the lipid mixture prior to liposome formation, a protocol that yielded over 80% encapsulation efficiency in both rigid and fluid liposomes. The presence of the active molecule did not modify the liposome size but increased the liposome negative charge and the liposome membrane fluidity, which suggested that the active molecule was accommodated in the lipid membrane. F_4t_-NeuroP integration in liposomes with a fluid character allowed for the selective targeting of the metastatic prostate cell line PC-3 vs. fibroblast controls. A significant decrease in viability (40%) was observed for the PC-3 cancer line in the presence of F_4t_-NeuroP fluid liposomes, whereas rigid F_4t_-NeuroP liposomes did not alter the PC-3 cell viability. These findings demonstrate that liposomes encapsulating F_4t_-NeuroP or other related molecules may be an interesting model of drug carriers based on membrane fluidity.

## 1. Introduction

### 1.1. Liposomes in Cancer Treatment

The latest sglobal data on cancer burden published by the World Health Organization show that in 2020, 10 million deaths were caused by cancer, which equals nearly one in six deaths. According to the International Agency for Research on Cancer (IARC), one of five people worldwide develop cancer during their lifetime. This alarming scenario highlights the need to find more effective treatments.

One major clinical concern is the lack of specificity of chemotherapeutic drugs [1]. Conventional chemotherapies have prominent side effects since molecules target not only tumors but also healthy tissues or induce systemic effects. At the end of the 19th century, the term magic bullet, introduced by Paul Ehrlich, led to the establishment of a new generation of cancer treatments. Originally, the concept was based on the selective targeting of a bacterium without side effects on other organisms caused by antitoxins or antibodies [2]. Over time, this concept led to the use of nanotransporters to selectively target cancer cells and allow a controlled release of active molecules. Among them, one can count inorganic nanoparticles (carbon nanotubes, iron oxide, mesoporous silica, gold nanoparticles, etc.), polymeric nanoparticles [3], dendrimers [4], micelles [5] or various lipid nanoparticles [6,7].

In this context, liposomes are one of the most studied drug delivery systems in the last 50 years. Their biocompatibility, biodegradability and low toxicity make them the ideal candidates for anticancer treatments. Their amphiphilic character allows for the encapsulation of a wide range of drugs: lipophilic molecules are located in the lipid bilayer, whereas hydrophilic molecules are dissolved in aqueous space [8]. The encapsulation of drugs in liposomes enhanced the therapeutic windows of different agents, reducing their pharmacokinetic and pharmacodynamic alterations [9]. Moreover, the lack of selectivity in vivo of some antitumoral drugs precludes their use in clinics [10]. Thus, their encapsulation considerably reduces undesirable side effects.

In addition, loaded drugs can be protected against inactivation, hydrolysis, early dilution in body fluids or environmental changes (temperature, pH) that could occur in vivo [11]. Since the approval of the first liposomal anticancer treatment, Doxil/Caelyx, in 1995 [3], liposomes have been considered one of the most successful nanoparticles in clinical cancer care as several liposomal preparations reached the market, such as Vyxeos in 2017, a prescription medicine used to treat acute myeloid leukemia [12], or Onivyde in 2015, for irinotecan delivery to the pancreas. Over the past three years, three liposomal products have been authorized by the FDA (U.S. Food and Drug Administration) and EMA (European Medicines Agency): Thermodox in 2021, thermally sensitive liposomes for the post-tumor ablation delivery of doxorubicin; Exparel in 2020, a local infiltration liposome indicated for post-surgical analgesia; and Arikayce in 2020, an oral inhalation treatment used for lung disease [13]. Even though the largest single application of lipid nanoparticles (LNPs), including liposomes, in drug delivery is cancer treatment, LNPs are also used to treat hormonal, respiratory or fungal diseases [14]. The last few years have been marked by the development of successful LNPs as delivery vehicles for nucleic acids in the two recently approved COVID-19 vaccines, Pfizer/BioNTech and Moderna [2].

### 1.2. Targeting Strategies: Significance of Membrane Fluidity

In the most currently used preparations, liposome accumulation in tumors is reached by passive targeting, but strategies based on the active targeting of tumors have also come to light. Cellular targets are receptors that are overexpressed in different cancer cells. That is why the surface of liposomes has been further ligated with small molecules, vitamins, proteins, antibodies, etc. [15]. Several targeted liposomal preparations have gone up to phase I or II clinical trials [16], such as transferin-targeted liposomes (MBP-426 from Mebiopharm or SGT-53 from SynergeneTherapeutics), HER-2-targeted liposomes (MM-302 from Merimack or C225_ILS-DOX from University Hospital, Basel) or GAH-targeted liposomes (MCC-465 from Mitsubishi Tanabe Pharma). Although clinical trials were discontinued for many, they are still ongoing for MBP-426 and C225_ILS-DOX. Yet, among the various immunoliposome types, only one preparation, Onivyde [14], is currently used in clinics. The scarce number of clinically available active targeting examples is mainly due to expensive antibody grafting strategies. Therefore, smaller molecules were proposed as active targeting strategies, such as RGD peptides [17] or folate [18], and some potential carriers are under clinical trials [19].

The process of tumorigenesis and tumor development is accompanied by many metabolic modifications. Several studies indicate that tumorigenesis is often accompanied by changes in membrane composition, which in turn determine membrane mechanical properties, i.e., membrane fluidity. Indeed, the relationship that may exist between the metastatic potential of cells and the alteration of their membrane fluidity has been evoked for many years [20]. In the case of breast cancer, for instance, cell malignancy was correlated with an increase in membrane fluidity (an increase in fluidity in the membrane of MT3 breast cancer cells correlates with enhanced cell adhesion in vitro and increased lung metastasis in NOD/SCID mice). This increase in the membrane fluidity of cancer cells can be explained by the alteration of the cell lipid metabolism. Cell membrane compaction is modulated by the presence of certain lipids, such as sphingolipids. The expression of *ceramide synthase-6* genes has been shown to decrease in tumor cells in contrast to non-tumor cells [21]. In the case of prostate cells, the membrane was less stiff, less viscous and thus more prone to deformation than that of the control cell line [22].

Although differences in the physicochemical properties of cell membranes between tumor and non-tumor cells have been reported over the years [21,23,24,25], they are not systematically used for the optimization of drug delivery strategies. In a previous study [26], we showed that targeting cancer cells based on the physical compatibility between cells and liposomes is a promising alternative targeting strategy. By modulating the lipid composition of liposomes, we associated their membrane fluidity state with that of the plasma membrane of targeted tumor cells. This match in membrane properties favors fusion processes between the liposome and the targeted membranes. Liposomes of controlled membrane fluidity were successfully used to deliver Monomethyl Auristatin E (MMAE) to the prostate tumor PC-3 cell line [27].

### 1.3. Oxylipins and Their Potential as Chemotherapeutics

In addition to the selective delivery of drugs to tumors, effective agents to treat this disease still need to be found. Natural products and their derivatives [28] have been used in cancer chemotherapy for over 50 years. No less than 49% of a total of 175 small-molecule anticancer drugs used in chemotherapy in Western countries over a 70-year period were obtained directly or derived from natural products [29].

Among the natural products found in our everyday diet, omega-3 and omega-6 polyunsaturated fatty acids (PUFAs) play important roles in human health as key precursors of many oxygenated metabolites called oxylipins [30]. PUFAs were first discovered in olive oil. Since then, numerous studies have shown the presence of these compounds and their derivatives in plants, cereals, rice and cocoa.

Oxylipins can be obtained through two pathways: an enzymatic pathway based on the catalysis of arachidonic acid (AA) by cyclooxygenase (COX), which leads to the formation of prostaglandins (PGs), or a non-enzymatic pathway initiated by free radicals. Discovered in 1990, non-enzymatic oxygenated PUFAs (NEO-PUFAs) have various effects in several biological mechanisms [30], suggesting medical potential. Due to the structure of these compounds being similar to that of PGs, they were named isoprostanoids (IsoPs). IsoPs are formed in the lipid membrane from phospholipids, and the molecules generated are racemic with a configuration of the two side chains, mainly *cis* [30]. The IsoP class includes molecules such as phytoprostanes (PhytoPs), neuroprostanes and isoprostanes. The NEO-PUFAs can be regarded as mediators of physiological and pathophysiological processes, including vasoconstriction, anti-arrhythmia, neuroprotection [30] and immunological responses [31].

More recently, it has been demonstrated that some PhytoPs reduce the viability of the MCF-7 and MDA-MB-231 breast cancer cell lines [28]. PhytoPs also possess activities such as anti-inflammatory and cell death-promoting activities and are also able to induce apoptosis in leukemia, Jurkat T-cells [32] and microglial cells [31].

In this study, we evaluated the cytotoxic effect of 12 free isoprostanoids, for which antitumor potential has been suggested but not validated yet. Among the 12 free isoprostanoids, one can find PhytoPs derived from α-linolenic acid (ALA), neuroprostanes (NeuroPs) derived from docosapentaenoic acid (DPAω3) or docosahexaenoic acid (DHA), and isoprostanes derived from arachidonic acid (AA). As the most promising cytotoxic effect was obtained for 4(*RS*)-4-F_4t_-neuroprostane (here abbreviated as F_4t_-NeuroP), this compound was encapsulated in liposomes to target cancer cells using the membrane-fluidity-based targeting approach previously described [26,27] from the perspective of the use of oxylipin in antitumor treatments for prostate cancer. The advantage of using this molecule is that the 4- and 20-series NeuroPs are the most abundant form of NeuroPs and are less prone to oxidation when compared with other PUFAs. Moreover, F4t-NeuroP has some anticancer properties. F4t-NeuroP showed antiproliferation effects on the human breast cancer cell line (MDA-MB-231), while no inhibitory effect was observed on a human mammary epithelial normal cell line (MCF-10A) [33]. The use of F4t-NeuroP in this study led to a good encapsulation rate (over 80%) of molecules inside the liposome and a notable cytotoxicity effect of the fluid liposome PO-F_4t_-NeuroP on the PC-3 metastatic prostate cell line. Altogether, in addition to their therapeutic potential, members of the NEO-PUFA family with antitumor activities may be efficiently encapsulated in selective carriers. Conventional chemotherapies have prominent side effects due to the non-specific targeting of cancer cells, which can affect healthy surrounding tissues but also induce systemic effects. The encapsulation of NEO-PUFA in liposomes may enhance the therapeutic window, increase efficacy and considerably reduce undesirable side effects.

## 2. Materials and Methods

The materials used for liposome preparation (lipids, polycarbonate membranes, extruder and syringes) were purchased from Avanti Polar Lipids (Alabaster, Al, USA). The materials used for the cell culture (Fetal Bovine Serum (FBS), Dulbecco’s Modified Eagle Medium (DMEM), Roswell Park Memorial Institute (RPMI) medium, phosphate buffered saline (PBS) pH 7.4), dimethyl sulfoxide (DMSO), calcein, paraformaldehyde (PFA) and resazurine) were purchased from Sigma-Aldrich (St. Louis, MO, USA).

### 2.1. Synthesis of F_4t_-NeuroP

The total synthesis of F_4t_-NeuroP was performed according to our published strategies [34,35].

**Scheme 1:**
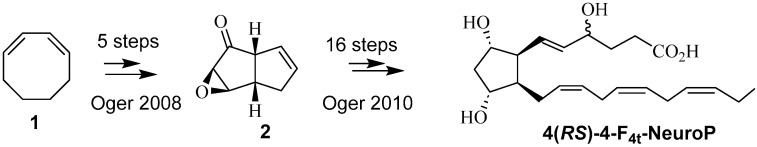
Synthesis of 4(*RS*)-4-F_4t_-NeuroP; details of the synthesis steps are provided in references [32,33].

The synthesis is summarized in Scheme 1. Starting from commercially available 1,3-cyclooctadiene 1, the key bicyclic intermediate 2 was obtained in 5 steps, giving an 18% yield. The introduction of α and ω chains was performed using regioselective protections/deprotections, oxidations and Wittig elongations. The final step was the saponification of the methyl esters in the presence of lithium hydroxide (LiOH) to obtain the free acid. 4(*RS*)-4-F_4t_-NeuroP was obtained from intermediate 2 in 16 steps with a 9% yield.

### 2.2. Liposome Preparation

Liposomes were prepared using the thin-film hydration method, followed by freeze/thaw cycles and extrusion. Then, 10 mg of lipids dissolved in chloroform were mixed in a round flask. Chloroform was evaporated under vacuum conditions on a rotatory evaporator at 50 °C to obtain a uniform lipid film, which was subsequently hydrated with 1 mL of PBS (10 mM phosphate, 137 mM NaCl and 2.7 mM KCl, pH 7.4) under stirring at 50 °C. To burst the MultiLamellar Vesicles (MLVs) formed, six freeze–thaw cycles in liquid nitrogen were applied to form Large Unilamellar Vesicles (LUVs). The LUVs were extruded through a 400 nm, followed by a 100 nm porous membrane with a Mini-Extruder (Avanti Polar Lipids, Alabaster, AL, USA) while being heated above their phase transition temperature (T_m_). The liposomes (10 mg/mL) were stored at 4 °C for 4 weeks without further extrusion.

F_4t_-NeuroP was added to the lipid mixture in chloroform prior to drying. Two liposomes with different membrane fluidities named PO (fluid) and DP (rigid) containing a final concentration of 200 µM F_4t_-NeuroP were prepared. This corresponded to 0.7% of the total lipid mass with a lipid/ F_4t_-NeuroP molar ratio of 69:1 at a molar percentage of 1.5%. PO liposomes were composed of POPC:DOPE:F_4t_-NeuroP (8:2:0.7 mole%), and DP liposomes were composed of DPPC:DOPE:F_4t_-NeuroP (8:2:0.7 mole%). The detailed liposome composition is given in Table 1. As the study is based on liposome cell interactions according to membrane fluidity, the test includes two liposomes with different physicochemical compositions: DP composed of long acyl chains (16:0), which do not allow movement within the membrane, and PO composed of acyl chains with unsaturations (16:0–18:1), which cause curvature and increase the membrane fluidity. Indeed, the physicochemical properties of liposomes are dictated by various acyl chain lengths and unsaturation. Long acyl chains are associated with a rigid membrane, and the presence of unsaturation increases fluidity. In a previous study [26], we showed that the interaction of the liposomes DO and PO’s preparations (fluid) with prostate cell lines, including PC-3, was modulated by their membrane fluidity. Conversely, none of the metastatic cell lines showed an interaction with the DP or DS preparations (rigid). Based on these results, we decided to use the PO and DP liposomes as carriers of opposite fluidity for the delivery of F_4t_-NeuroP and to check whether the match between carrier and target cell membrane fluidity permits the selective delivery of the active principle. Such biocompatible, biodegradable carriers offer a cost-effective targeting strategy with respect to antibody or molecule grafting.

Excess/free F_4t_-NeuroP was removed using steric exclusion chromatography (SEC, PD-10 columns, Sephadex^TM^ G-25 M, GE Healthcare (Limonest, France). In brief, the column was equilibrated with PBS, and then 1 mL of liposome preparation was added, and the samples were eluted with PBS. As F_4t_-NeuroP showed a maximum absorbance at 240 nm, the absorbance was measured at this λ for each fraction, and the amount of F_4t_-NeuroP was estimated against an F_4t_-NeuroP standard curve.

Fractions of 1 mL were recovered, and the 240 nm absorbance was measured. As the liposomal solution exhibited a strong diffusion, the amount of encapsulated F_4t_-NeuroP was estimated as the total F_4t_-NeuroP added minus the non-encapsulated F_4t_-NeuroP. All measurements were performed in triplicate. The encapsulation efficiency (EE) and drug loading (DL) were calculated following Equations (1) and (2).

(1) $$EE\left(\%\right)=\frac{Total\;F4t-NeuroP\;concentration-F4t-NeuroP\;free\;molecule\;}{Total\;F4t-NeuroP\;concentration}\times 100$$

(2) $$DL\;\left(\%\right)=\frac{Amount\;of\;F4t-NeuroP\;in\;liposomes}{Amount\;of\;liposomes}\times 100$$

### 2.3. Physico-Chemical Characterization of Blank and F_4t_-NeuroP-Loaded Liposomes

The membrane fluidity of liposomes was measured using radiometric probes and fluorescence spectroscopy on a FP-8500 spectrofluorimeter (JASCO Applied Science, Halifax, Canada). Liposomes at a concentration of 0.1 g/L were incubated with the probe Dioll at 0.4 µM for 1 h. The fluorescence emission spectrum was recorded from 400 nm to 600 nm with an excitation wavelength of 390 nm. Emission and excitation slits were set at 2.5 nm. Experiments were performed at 37 °C. The Generalized Polarization (GP) parameter was calculated [36,37], as indicated in Equation (3),

GP = (I_440_ − I_490_)/(I_440_ + I_490_) (3)

where I_440_ is the fluorescence emission intensity at 440 nm (gel phase) and I_490_ is the fluorescence emission intensity at 490 nm (liquid crystalline phase).

The Z-average diameter (the intensity-weighted mean hydrodynamic size) and polydispersity index (PDI) of each liposome preparation were determined for each preparation with and without F_4t_-NeuroP. The liposomes were diluted at a concentration of 0.1 mg/mL in PBS. The analyses were carried out at 25 °C with an angle of detection of 173°.

The surface charge of the liposomes was obtained by measuring the ζ potential values obtained from the electrophoretic mobility of the liposomes in dispersion using Malvern Zetasizer^®^ Nano ZS (Malvern Instruments S.A., Worcestershire, UK). The liposome preparation was diluted to 1/10 with water to decrease the solvent ionic force and ensure that the solvent used does not interfere with the measurement of the liposome charge.

The results were expressed as the mean ± standard deviation of three independent liposome preparations.

### 2.4. Cell Culture

The PC-3 cell line was purchased from ATCC (Manassas, VA, USA). This cell line was originally isolated from a vertebral metastasis stemming from a prostate tumor and entirely composed of carcinoma cells [38]. The fibroblasts used are primary cells from the dermis, and more specifically, foreskin cells. This cell line was purchased from the hospital cell and tissue bank (HCL, Lyon, France). Fibroblasts and PC-3 cells were cultured in a DMEM medium supplemented with 10% (*v*/*v*) FBS, 100 U/mL of penicillin and 100 µg/mL of streptomycin. PC-3 cells were used because of their high metastatic potential (grade IV prostate cancer), and fibroblasts were used because they are considered a classical control line, making them a valuable model of healthy tissue. All cells were cultured in a humidified incubator at 37 °C with 5% CO_2_. After standard trypsinization, 78,000 cells of PC-3 and 25,000 cells of fibroblast per well (500 µL) were seeded in 24-well plates and incubated overnight before treatment with the different drug candidates. These cell densities were chosen to obtain a surface coverage of 80% after 72 h of culture. The cell passages for optimal conditions for culture are P5 for fibroblast and P15 for PC-3.

For cytotoxicity experiments with free isoprostanoids, a concentration of 100 µM of each of the 12 molecules was added to the cell culture medium. Tests with liposomes encapsulating F_4t_-NeuroP were performed with a final concentration of 50 µM F_4t_-NeuroP in the medium. This corresponded to a lipid concentration of 2.5 mg/mL.

After 5 h of contact with the different molecules, the plates were washed with PBS and cultured in a fresh medium. The plates were then incubated again for 72 h at 37 °C in a humidified incubator with 5% CO_2_ before performing the cell viability measurements.

### 2.5. Cell Viability Assay

The cell viability measurements were performed using the resazurin assay. This method is based on the assessment of the metabolic capacity of the cell population after drug exposure. It is based on the conversion of Alamar blue (resazurin) to a resorufin compound in living cells. Resazurin can pass through the cell membrane into the cell, where it is reduced and transformed into a pink, fluorescent compound that is called resorufin. Dead cells that cannot reduce resazurin because they lack metabolic activity will not be able to generate a fluorescent signal [39]. This viability assay is a rapid and sensitive measurement of the metabolic activity of cells. The compound used for this viability test, Alamar blue, does not degrade exposed cells. The advantage of using this non-toxic compound is that it allows the cells to be re-cultured or other tests to be carried out in parallel [40].

A culture medium containing resazurin at a concentration of 0.03 mg/mL was prepared and preheated at 37 °C. The culture medium was removed, and the plates were rinsed with warm PBS. Then, 300 µL of a warm resazurin medium was added and incubated at 37 °C, with 5% CO_2_ for 40 min. Finally, in a 96-well black-bottom plate, 200 µL of each solution was added, and the fluorescence (excitation at 550 nm and emission at 590 nm) was read on an Infinite-M200 pro Plate reader (TECAN, Männedorf, Switzerland). The activity of the cells treated with Triton corresponded to 0% viability, and the untreated control corresponded to 100% viability. The results were expressed as the mean ± standard deviation of three independent experiments, and the percentage of viability was calculated as indicated in Equation (4).

(4) $$\%\;\mathrm{cells}\;\mathrm{viability}=\frac{IntensityF_{Sample}-IntensityF_{Triton\;}}{IntensityF_{Control}-IntensityF_{Triton}}$$

### 2.6. Statistical Analysis

All tests were performed in triplicate (n = 3). Thus, from the data, we calculated the standard deviation (SD) corresponding to the average amount of variability in the dataset. The results are expressed as the mean +/− SD of three independent replicates.

Student’s *t* test was also carried out to compare the means of two samples. *** *p* < 0.001; ** *p* < 0.01; and *p* < 0.05.

## 3. Results

### 3.1. Cytotoxic Effect of 12 Free Isoprostanoids

**Figure 1:**
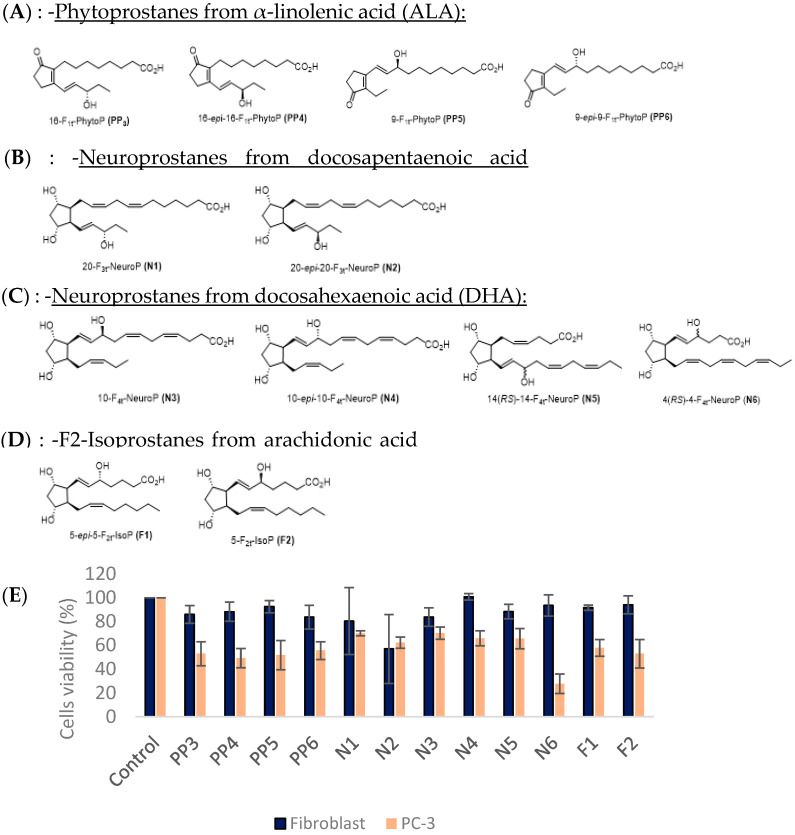
Structures and names of the 12 isoprostanoids (**A**–**D**). Cell viability of the PC-3 (salmon) and fibroblast (dark blue) lines in the presence of 100 µM of PP3, PP4, PP5, PP6, N1, N2, N3, N4, N5, N6, F1 and F2 (**E**). Cell viability was assessed as metabolic residual activity tested with resazurin assay. Results are expressed as mean ± SD of three independent replicates.

First, a comparative study on several isoprostanoids was performed to identify effective antitumor molecules on different cancer cell lines. In previous studies, several classes of oxylipins derived from different polyunsaturated fatty acids may have been attributed with potential antitumor activity. For instance, PhytoPs have revealed their cytotoxic effect on breast cancer cells [28]. In the present report, we carried out experiments on 12 isoprostanoids, including PhytoPs (Figure 1A), NeuroPs derived from docosepentaenoic acid (Figure 1B), NeuroPs derived from docosahexaenoic acid (Figure 1C) and IsoPs (Figure 1D), and measured their cytotoxic effect on PC-3, a highly aggressive prostate cancer line, and fibroblasts, a non-tumoral cell line (Figure 1E).

The isoprostanoid sensitivity of the different cell lines was measured by estimating cell metabolic activity using a resazurin assay as a marker of cell viability. Cells were exposed for 5 h to a concentration of 100 µM of each isoprostanoid dissolved in DMSO. After 5 h, the cells were washed with PBS to remove all liposomes that had not fused with the cells and then incubated again for 72 h to allow the drug to act on the cell metabolism. Then, the medium was removed, and the cells were incubated with a fresh medium containing resazurin. Depending on the percentage of metabolically active cells, resazurin is transformed into a blue-colored compound, resorufin. The absorbance was measured, and a cell viability % was calculated with respect to the control.

The presence of isoprostanoids did not affect the viability of the fibroblasts, except for the N2 sample (approximately a 40% decrease in viability). A decrease of at least 30% of the viability of the PC-3 prostate cancer cells was observed in the presence of all the molecules tested. The most significant decrease in PC-3 cell viability was observed in the presence of the N6 molecule: over a 70% loss in viability for the cancer line. N6, further denoted as F4t-NeuroP, was selected for liposome encapsulation. F4t-NeuroP is a derivative of docosahexaenoic acid (DHA). Over the past few years, DHA has been shown to be implicated in numerous biological mechanisms and have cardioprotective or anti-arrhythmic properties. Moreover, it has already been established that F4t-NeuroP is implicated in molecular mechanisms such as the protection of the ryanodine receptor, a form of intracellular calcium channels implicated, for example, in colorectal cancer metastasis [41], or induces antiproliferative effects in the human breast cancer cell line (MDA-MB-231) [28].

### 3.2. Encapsulation of F4t-NeuroP in Liposomes

Once the cytotoxic activity of F4t-NeuroP was evaluated in its soluble form, this compound was encapsulated in liposomes of a distinct membrane fluidity.

Membrane fluidity is related to the motional capacity of membrane components. This is dictated by lipid chains and their capacity to interact with each other. Therefore, the membrane fluidity was modulated according to the nature of the acyl chains (length) and/or the number of unsaturations of the phospholipids that constituted the liposome membranes. Based on previously published studies [26,27], two liposome samples with different membrane fluidities were prepared: a rigid one composed of 1,2-dipalmitoyl-sn-glycero-3-phosphatidylcholine (DPPC), further denoted as DP, and a fluid one composed of 1-pamitoyl-2-oleoyl-sn-glycero-3-phosphatidylcholine (POPC), further denoted as PO. DPPC, which possess long saturated acyl chains (16:0), forms rather rigid membranes at 37 °C. The order/disordered transition temperature for DPPC membranes is 40 °C. This means that at the working temperature, membranes composed of DPPC are in a rigid/ordered state. On the other hand, PO liposomes are composed of 80% POPC, which contains one saturated (C16:0) and one unsaturated (C18:1) acyl chain. The cis double bond induces chain torsion and limits the possibility of interactions between lipid molecules in the bilayer. Therefore, the order/disordered transition temperature for POPC membranes is 4 °C, and at 37 °C, the liposome membrane becomes fluid. In addition, 20% of a fusogenic lipid was added to promote inverted hexagonal phase intermediates that favor membrane fusion, as described in the Materials and Methods Section 2.

F4t-NeuroP was dissolved in chloroform and added to the lipid mixture at a final concentration of 200 µM for a lipid concentration of 10 mg/mL (lipid/drug molar ratio of 69:1). The chloroform was evaporated, and the lipid-F4t-NeuroP film was resuspended in PBS. The liposomes were prepared as described in the Materials and Methods section with a conventional freeze/thaw protocol, followed by an extrusion at 100 nm. The different liposome compositions are listed in Table 1 (Materials and Methods). Free F4t-NeuroP was removed with SEC.

**Figure 2:**
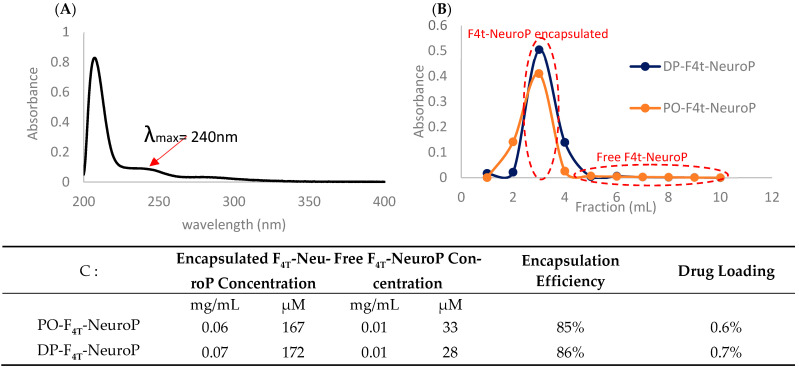
Absorbance spectrum of F4t-NeuroP in the UV region (**A**). Elution profile of PO and DP F4t-NeuroP from SEC column: absorbance at 240 nm vs. elution volume (**B**). Encapsulated and free F4t-NeuroP concentrations (**C**).

The UV spectrum of the F4t-NeuroP dissolved in PBS showed an absorbance shoulder at 240 nm (Figure 2A). Therefore, the absorbance of the SEC elution fractions was measured at this wavelength, and the F4t-NeuroP elution profile was plotted (Figure 2B). A major elution peak was obtained at around a 3 mL elution volume, which roughly corresponded to the column void volume. This peak was attributed to the elution of the liposome-containing fraction. The free F4t-NeuroP was eluted in 5 to 10 mL fractions. Using a standard calibration curve of the F4t-NeuroP dissolved in the buffer, we calculated the F4t-NeuroP concentration of each sample. Since the liposome-containing fractions presented a high turbidity due to liposome particle light diffusion, the encapsulation efficiency (%) was calculated by subtracting the total amount of F4t-NeuroP added before the liposome preparation from the free F4t-NeuroP (fractions 5 to 10).

The results show approximately 85% and 86% encapsulation efficiency for the samples PO-F4t-NeuroP and DP-F4t-NeuroP, respectively (Figure 2C).

### 3.3. Liposome Characterization

**Figure 3:**
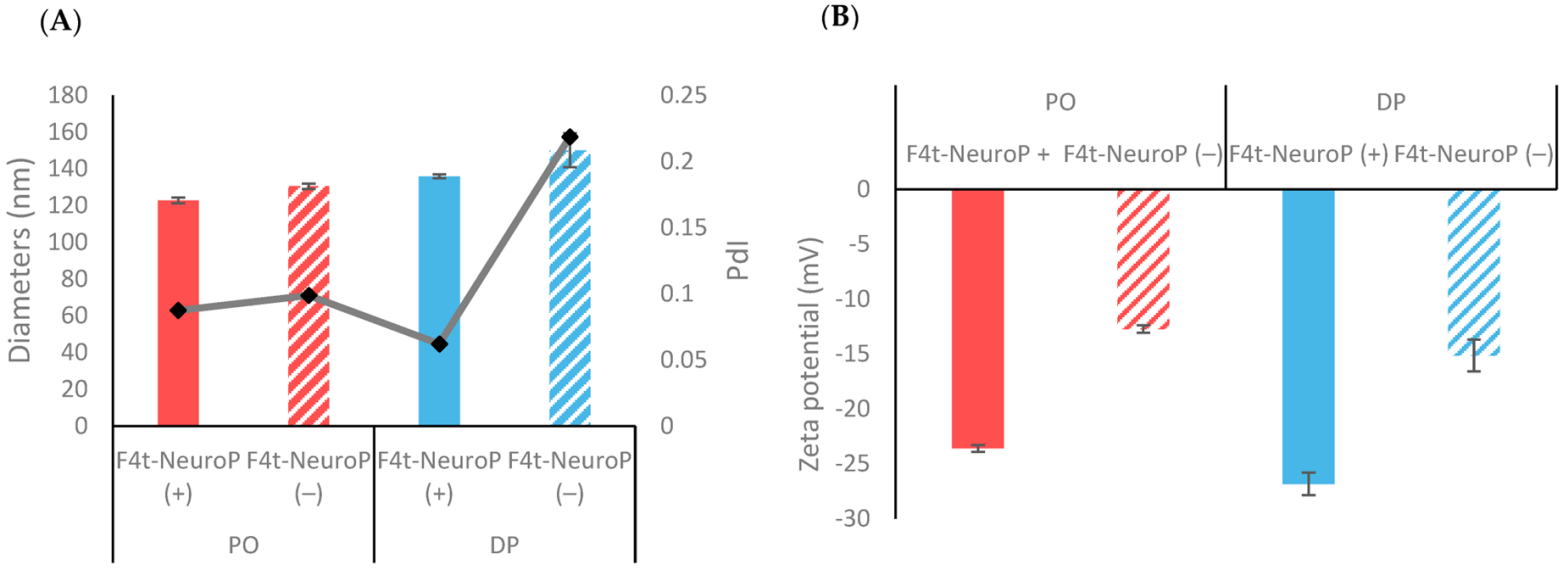
Liposome average size, polydispersity index (PDI) (**A**) and zeta potential (**B**) in the absence or presence of F4t-NeuroP. Results are expressed as mean ± SD of three replicates.

To assess the physico-chemical properties of the liposomal suspension for in vivo administration, the size and the polydispersity of the liposomes containing or not containing F4t-NeuroP were measured via dynamic light scattering (Figure 3A).

The F4t-NeuroP liposomes had a diameter of about 150 nm with a PdI varying between 0.05 and 0.2. These results suggest that the size of the PO and DP liposomes was uniform, close to the extrusion size, and that the preparations are monodisperse with a PdI < 0.2. No significant change in the liposome diameter was observed with or without F4t-NeuroP. In terms of charge, the addition of F4t-NeuroP increased the overall charge of both liposome preparations, −24 mV for PO-F4t-NeuoP and −27 mV for DP-F4t-NeuroP (Figure 3B). This can be explained by the insertion of F4t-NeuroP into the bilayer of the liposomes, exposing its negative charge to the solvent.

### 3.4. Liposome Membrane Fluidity Assessment

**Figure 4:**
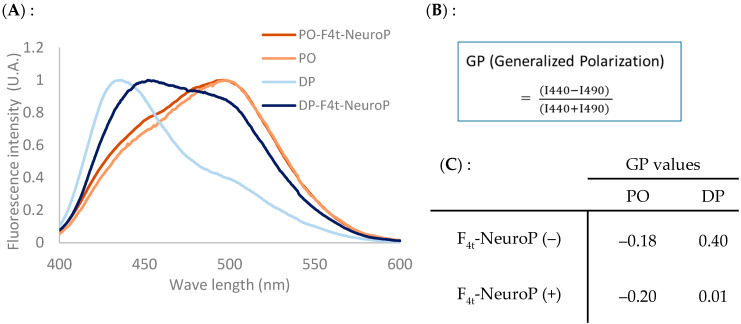
Evaluation of liposome membrane fluidity. Fluorescence emission spectra of Dioll inserted in liposomes containing or not containing F_4t_-NeuroP at 37 °C (λexc of 390 nm) (**A**). GP formula (**B**) and values calculated for each liposome composition: top—liposomes without F4t-NeuroP, bottom—F_4t_-NeuroP-containing liposomes (**C**).

As previously reported, the interaction of liposomes with cancer lines can be modulated according to the carrier’s membrane fluidity. To check if the addition of the F_4t_-NeuroP derivative alters the liposome membrane fluidity, this parameter was measured using a fluorescent probe, namely Dioll, that is spontaneously inserted in the bilayer, and its fluorescence emission is related to the change in viscosity of its environment. The fluorescence emission spectra (from 400 to 600 nm) of the liposomal preparations containing F_4t_-NeuroP obtained with Dioll at 37 °C with an λex of 390 nm are shown in Figure 4A.

For the PO fluid liposomes, the Dioll spectrum has a predominant peak at 490 nm, characteristic of a liquid crystalline state. In contrast, the rigid DP liposomes exhibit a Dioll spectrum with a predominant peak at 440 nm. The presence of F_4t_-NeuroP changes this profile with an increase in the fluid phase contribution. This modification is particularly important for the DP-F_4t_-NeuroP preparation. In this case, two peaks (440 and 490 nm) of nearly equal contributions are observed.

The GP parameter, as the numerical index of membrane fluidity, was calculated from the fluorescence emission spectra described above (Figure 4B). Both the PO and PO- F4t-NeuroP liposomes have membranes in a fluid state, as shown by the negative GP values of −0.18 and −0.20, respectively. The DP liposomes have a high GP of 0.4 in the absence of F_4t_-NeuroP, characteristic of a rigid membrane state. This value strongly decreases for the DP- F_4t_-NeuroP preparation with a GP of 0.01.

A comparison of these GP values with those of the liposomes lacking F_4t_-NeuroP shows that the addition of F_4t_-NeuroP increases the overall fluidity of liposomes, with the GP value being lower in the presence of F_4t_-NeuroP than in its absence, with a most prominent effect on the rigid DP liposomes. It can be assumed that F_4t_-NeuroP is inserted into the membrane of liposomes between the acyl chains, thus decreasing their ordering capacity. Since the active molecule is capable of modifying the liposome fluidity, it is important to measure it in a systematic way.

### 3.5. Cytotoxicity Effect of F_4t_-NeuroP Liposomes on PC-3 Prostate Tumor Cells

**Figure 5:**
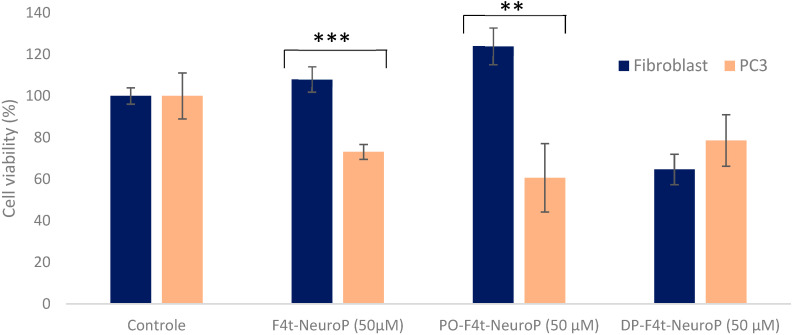
Cellular viability of fibroblasts and PC-3 prostate cancer lines (grade IV bone metastasis) in the presence of 50 µM of F4t-NeuroP and in the presence of 50 µM of liposomes—F_4t_-NeuroP. Results are expressed as mean ± SD of three independent replicates. *** *p* < 0.001; ** *p* < 0.01; Student test.

Finally, the effect of F_4t_-NeuroP encapsulated in liposomes was evaluated by a test of cytotoxicity on a prostate cancer cell line using membrane fluidity as a physicochemical parameter. The cells used were of the PC-3 human prostate cell line derived from bone metastasis and of high tumor aggressiveness. In parallel, a fibroblast cell line was used as a non-tumoral control. The results are presented in Figure 5.

The inhibitory effect of free F_4t_-NeuroP was tested at a concentration of 50 µM in DMSO on the fibroblast and PC-3 lines. The viability of the PC-3 cancer lines decreased by 25% in the presence of 50 µM of DMSO with respect to the untreated control. Conversely, the fibroblast viability was not affected by the presence of F_4t_-NeuroP.

The effect of the PO and DP- F_4t_-NeuroP encapsulated on the PC-3 and fibroblast cell lines was tested at a final concentration of 50 µM of F_4t_-NeuroP. The effect on cell viability is highly dependent on the type of liposome and, more especially, on the liposomal membrane fluidity. Rigid DP-F_4t_-NeuroP liposomes have a reduced efficiency on the cancer cell line (80% of viability remaining) and a higher efficiency on fibroblasts (65% of viability remaining). For fluid liposomes, PO-F_4t_-NeuroP liposomes have the opposite behavior, with 60% remaining viability for PC-3 and 120% for fibroblasts. These results are consistent with previous studies revealing that liposomes with fluid membranes fuse more easily with cancer cells [27]. Moreover, the encapsulation of F_4t_-NeuroP in the PO liposomes further increased the effect on cell metabolic activity with respect to the free molecules at a similar concentration of 50 µM.

## 4. Discussion

In vivo, most oxylipins are biosynthesized by enzymes such as cyclooxygenase. Yet, in 1990, a new class of oxylipins was described, namely the non-enzymatically oxygenated polyunsaturated fatty acids (NEO-PUFAs) [42]. NEO-PUFAs are mediators of physiological and pathophysiological processes such as vasoconstriction, anti-arrhythmic processes and cell proliferation.

The therapeutic potential of oxylipins has been the subject of numerous studies, and applications have been found in the treatment of Parkinson’s disease (Apokyn^®^), hereditary tyrosinemia (Orphadin^®^) and cancers [43]. In the present work, 12 non-enzymatically oxidized oxylipins, termed isoprostanoids, were tested to study their potential anticancer activity. Their effect on cell metabolic activity was tested on the highly aggressive PC-3 cancer cell line. All the molecules tested showed an effect on the cancer cell metabolic activity. Therefore, this class of oxylipins may have therapeutic potential either alone or in conjunction with other chemostatics [28]. Of note, all the isoprostanoids tested, except one, show little effect on fibroblasts, which may suggest some extent of tumor selectivity.

F_4t_-NeuroP was the molecule with the most important inhibitory action on PC-3. Based on the concept of the membrane physicochemical properties of liposomes as a means for specific drug delivery, we encapsulated F_4t_-NeuroP in two liposome samples of opposite membrane fluidities, the PO fluid and DP rigid samples.

The use of liposomes as drug delivery nanosystems allows for the encapsulation of a wide range of molecules (hydrophobic and hydrophilic) with therapeutic potential activities. There are two modes for the incorporation of molecules into liposomes: a so-called passive encapsulation, where the hydrophilic molecules are dissolved in the aqueous phase used to hydrate the lipid film and the hydrophobic molecules are dissolved in the organic solvent; and an active encapsulation, where the use of a pH gradient is sometimes necessary to encapsulate hydrophilic molecules with protonable chemical functions [44]. F_4t_-NeuroP was introduced in the lipid mixture prior to solvent evaporation. After the liposome formation process and the removal of non-encapsulated molecules, an evaluation of the encapsulation efficacy was performed. Good encapsulation yields were systematically obtained: 85% of the active molecule was systematically associated with the liposomal fraction, whatever the phospholipid composition of the liposomes.

**Figure 6:**
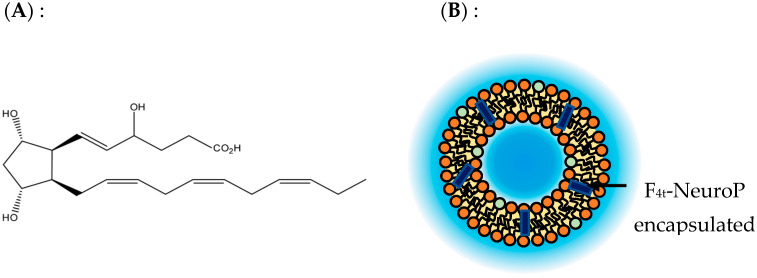
Structure of F_4t_-NeuroP (**A**). Schematic representation of a liposome encapsulating neuroprostane (**B**).

Before testing the inhibitory action of the liposomes loaded with F_4t_-NeuroP, these liposomes were characterized in terms of their size, PdI, zeta potential and membrane fluidity with a view to their use for in vivo administration. The addition of F_4t_-NeuroP does not influence the average size of the liposomes or their dispersity. In terms of charge, the addition of F_4t_-NeuroP increases the overall negative charge of the liposomes. Of note, F_4t_-NeuroP possesses a negative charge at a pH of 7.4 due to the presence of a carboxylate group (Figure 6). By inserting it into the liposome membrane, F_4t_-NeuroP probably gives them negative charges. We can therefore assume that F_4t_-NeuroP is able to be accommodated in the membrane of the lipossome between lipids.

This assumption is further supported by the increased membrane fluidity of the liposomes prepared in the presence of F_4t_-NeuroP-containing preparations. This increase, limited in the case of fluid PO preparation, is particularly important in the case of rigid DP preparation. In this case, the insertion of F_4t_-NeuroP between lipids most probably hinders chain ordering in the DP membrane of liposomes, resulting in increased membrane fluidity.

It is important to note that the presence of charges in liposomes (positive or negative) reduces the aggregation phenomena between liposomes thanks to the increase in the electrostatic repulsive forces between them. Moreover, the charge of liposomes has a preponderant impact on their recognition by opsonins in the bloodstream. Particles with too-high positive or negative charges will be eliminated more quickly than uncharged particles [45]. It is thus necessary to obtain a composition sufficiently charged to be stable but not excessively charged to avoid in vivo recognition by opsonins and rapid clearance from the body. Therefore, the moderate negative charge of PO-F_4t_-NeuroP liposomes is highly compatibility with an in vivo application.

The resazurin viability assays showed the specificity of the action of PO-F_4t_-NeuroP on PC-3 cells, with a remaining viability of 40%. This notable effect confirms that liposomes with fluid membranes specifically fuse with cancer cells [26,27]. According to the literature, cancer cells have a modified metabolism compared to normal cells. Indeed, tumor cells present an alteration of their lipid profile [21] that is expressed by an increase in unsaturated fatty acid levels and thus makes the cell membrane more fluid [46]. Thus, fibroblasts are expected to be globally more rigid, whereas PC-3 cells have a metastatic tumor cell line and are globally more fluid. Membrane fluidity determines mobility of the lipids, proteins and water molecules that cooperate in the reorganization and assembly required and induced by the membrane fusion [47,48]. Thus, modulating the lipid composition of liposomes could be an effective and specific therapeutic agent to target cancer cells and to exploit the general tendency toward an increased membrane fluidity in tumor cells. The correlation in membrane fluidity between the liposomes and the targeted cancer cell will facilitate membrane fusion and thus the release of the molecule of interest into the target cell.

At this point, it is difficult to assess the cellular target of the molecules. Cancer cells can exhibit properties different from fibroblasts or other non-tumor cells such as proliferative signaling, an evasion of growth suppressors, a resistance to apoptosis, replicative immortality, copious angiogenesis, active invasion and metastasis. Several parented molecules target apoptotic mechanisms, but a further analysis is needed to reveal oxylipin cellular targets.

Alternatively, several studies show that prostate cancer cells and PC-3 cells, in particular, show different lipid profiles [49,50]. Moreover, in a previous study, we showed that the membrane and cell viscosity of PC-3 were different from those of the non-tumor control [22]. We can argue that the PC-3 membrane may be more loosely packed than that of fibroblasts. Therefore, hydrophobic molecules such as F4t-NeuroP may enter the PC-3 membrane, but not that of fibroblasts, and thus exert a differential action.

This hypothesis is supported by the experimental results from Figure 4 in the manuscript, which show an effect of F_4t_-NeuroP on fibroblasts presented to cells in its encapsulated DP form, a rigid type of liposomes that are uptaken by this type of cell.

This intrinsic specificity may be increased by the use of an appropriate delivery system. In this report, the selective drug delivery is based on the correlation between the fluidity of the cell and that of the liposome membrane. The similarity in membrane fluidity between the PO liposomes and PC-3 cancer cells may facilitate membrane fusion and preferential targeting by the fluid liposomes.

Altogether, by showing a cytotoxic effect on PC-3, a highly aggressive tumor line, F_4t_-NeuroP may open the way for new derivatives of therapeutic interest. This family of molecules may provide a structural basis for the conception of new antitumor drugs. In the present report, we show that F_4t_-NeuroP can be easily encapsulated in liposomes, with the PO-F_4t_-NeuroP liposome representing a promising strategy to promote specific drug delivery and potentiate antitumor activity. PO-F_4t_-NeuroP liposomes constitute an interesting example of drug carriers, with low manufacturing costs, simple preparation protocols and a proven specificity of action on cancer cells. Based on these encouraging results, studies on biodistribution, stability and tumor accumulation, as well as storage stability and the drug release rate, must be carried out to confirm the translational potential of the preparation of these liposomes.

## Figures and Tables

**Table 1:** Lipids used for liposome preparation: name, fatty acid composition, structure and phase transition temperatures (melting temperature (Tm)).

Molar%	Liposome Preparation	Acyl Chain Composition	Lipid Name and Structure	T_m_
Main lipid (80%)	PO	16:0-18:1 PC	POPC 1-palmitoyl-2-oleoyl-glycero-3-phosphocholine 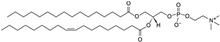	−4 °C
	DP	16:0 PC	DPPC 1,2-dipalmitoyl-glycero-3-phosphocholine 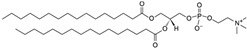	41 °C
Fusogenic lipid (20%)	All preparations	18:1 (Δ9-Cis) PE	DOPE 1,2-dioleoyl-sn-glycero-3-phosphoethanolamine 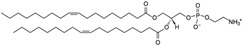	−16 °C
4(RS)-4-F4t-Neuroprostane (0.7% on total lipid mass)	In two preparations		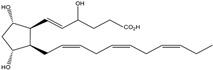	

## Data Availability

The data can be shared up on request.
